# Some considerations on target estimands for health technology assessment

**DOI:** 10.1002/sim.9566

**Published:** 2022-11-17

**Authors:** Antonio Remiro‐Azócar

**Affiliations:** ^1^ Medical Affairs Statistics Bayer plc Reading UK

First and foremost, I would like to thank an anonymous Associate Editor and Prof. Nigel Stallard for arranging a fascinating discussion around my article, “Target estimands for population‐adjusted indirect comparisons.”^1^ This is based on a prior exchange with Phillippo et al.^2^, ^3^, ^4^ I extend my gratitude to Russek‐Cohen,^5^ Schiel,^6^ Senn,^7^ Spieker,^8^ and Van Lancker et al,^9^ for their additional contributions.

This rejoinder discusses the potential development of an estimands framework in the context of evidence synthesis and health technology assessment (HTA). I consider the following base‐case scenario. An evidence synthesis (eg, an indirect treatment comparison or a network meta‐analysis) is required for HTA. The evidence synthesis combines the results of multiple randomized controlled trials (RCTs).^10^, ^11^, ^12^ Each RCT has been designed for regulatory approval in the premarketing authorization setting, and has target estimands of its own. There are exceptions to this scenario; regulatory decisions are not exclusively based on RCTs and HTA decisions are not exclusively based on evidence synthesis. Nevertheless, it is a common scenario.

Two challenges arise. First, different policy decisions will require different research questions; the estimands targeted for regulatory approval will likely not align with those targeted for HTA. Second, within the evidence synthesis, there may be misalignment between the estimands targeted by individual studies. Finally, I reassert that marginal estimands should be preferred in the context of the original discussion.^2^, ^3^, ^4^

## 1 HARMONIZATION BETWEEN DECISION‐MAKING CONTEXTS

For RCTs in the regulatory setting, the use of estimands has been stimulated by the recent publication of the E9 (R1) Addendum, issued by the International Council of Harmonisation (ICH) and recognized by agencies such as the United States Food and Drug Administration.^13^ According to the ICH guidance, an estimand should be defined on the basis of five attributes: (1) treatment(s); (2) target population; (3) clinical outcome of interest; (4) population‐level summary effect measure; and (5) strategy for intercurrent (post‐randomization) events. My original commentary^1^ places focus on the second and fourth elements. It does not intend to cover other dimensions, which are also important. The application of the ICH guidance to individual RCTs has been addressed in depth elsewhere.^14^, ^15^

In evidence synthesis, and more generally in HTA reporting, the term *estimand* is rarely used. This does not imply a lack of interest in clarifying the research question prior to the analysis. In HTA, the PICO framework is typically used to translate policy questions into research questions. PICO consists of four components: (1) population; (2) intervention; (3) comparator(s); and (4) outcome. To obtain reimbursement for a given treatment, the manufacturer must submit an evidence dossier that addresses the research question(s) included in the scope. Current best‐practice guidelines recommend prespecifying the relevant PICO question(s) in the HTA scoping process.^16^, ^17^, ^18^ In evidence synthesis, PICO questions are often formulated prior to the analysis in order to guide the data extraction required for systematic literature reviews.^19^

The PICO framework is a valuable tool. Nevertheless, it needs refinement to more precisely characterize the inferential target in HTA; that is, to map the HTA research question into a formal estimand. The current framework does not discuss the population‐level measure used to summarize the treatment effect. It also lacks a strategy for intercurrent events, which as stated by Van Lancker et al,^9^ “are at the heart of the ICH E9 (R1) addendum.” I would propose a PICOSI framework for describing the HTA research question: (1) population; (2) intervention; (3) comparator(s); (4) outcome; (5) summary effect measure; and (6) intercurrent events. This would make the summary measure used to compare treatments and the intercurrent event strategy explicit. It would also allow stakeholders to translate regulatory language to HTA terminology.

My original commentary^1^ and prior exchange with Phillippo et al^2^, ^3^, ^4^ highlight the importance of prespecifying the summary effect measure. It is necessary to make a distinction between marginal and conditional effect measures. I argue that the former are more relevant to the HTA setting.^1^, ^3^ Another question that arises is whether relative estimands (treatment effects) or absolute estimands (mean outcomes) are more appropriate. When the goal is to estimate comparative effectiveness, relative estimands are the logical choice. Nevertheless, when the aim is to estimate comparative cost‐effectiveness, absolute measures (eg, event probabilities) are often input to health economic models.^4^ The transportability of relative and absolute measures between populations depends on different conditions.^20^ Another important matter is that the evaluation of effect modifier status, necessary to assess generalizability, transportability and external validity, is tied to the scale used to measure the relative effect.^21^ Effect modifiers cannot be selected prior to the specification of a summary effect measure of interest.

Fundamentally, the estimands required for regulatory and HTA decision‐making are expected to diverge. Regulatory decisions are made prior to HTA decisions. Individual studies will likely be completed at the time of undertaking the evidence synthesis, and estimand definitions for the former will precede estimand definitions for the latter.

Moreover, regulatory and HTA decision‐makers have different perspectives. The HTA paradigm places emphasis on evaluating “real‐world” effectiveness, whereas regulators typically prioritize internal over external validity when assessing clinical efficacy. In terms of intercurrent events, estimands based on the “treatment policy” and the “composite” strategies seem more relevant for HTA purposes. Nevertheless, regulators may prefer other strategies in certain scenarios. In terms of comparator(s), HTA demands comparing the intervention under study versus all alternative treatments for a specific disease state. Conversely, regulators typically require a head‐to‐head comparison against placebo or standard of care.

In addition, there is a multiplicity of decision‐makers within HTA. Different agencies will target different populations and, therefore, different estimands. HTA policy decisions typically consider external validity, but external validity depends on the population that is targeted by the payer. Given the large number of regulatory bodies and national and regional HTA agencies, I coincide with Schiel: we should avoid focusing exclusively on the viewpoint of a single stakeholder when designing studies.^6^ Traditionally, HTA objectives have played a limited role in the design of RCTs. Potential HTA research questions should be posed in the early stages of drug development, so that these can be answered at the time of HTA decision‐making.^22^, ^23^

## 2 STANDARDIZATION WITHIN THE EVIDENCE SYNTHESIS

Apart from mismatches in estimands between decision‐making contexts, there may also be inconsistencies between the trial‐specific estimands combined in an evidence synthesis. As expressed by Russek‐Cohen, “individual studies were not planned with similar estimands nor they were necessarily planned in anticipation of a meta‐analysis.”^5^ There may be mismatches in endpoint definitions, treatment implementations, study target populations, summary effect measures or intercurrent event strategies, that invalidate the results of an evidence synthesis.

The availability of standardized outcome and treatment definitions depends on the clinical indication and the interventions being evaluated. Differences in endpoints may arise if outcomes are measured using different methods, at different times, or if the trials have different duration. When a treatment is assessed in multiple studies, there may be dissimilarities between the versions of the intervention being administered, for example, in dosing formulation, delivery mechanism, co‐treatment regimens, or concomitant background medications. Clinical input is critical to assess the potential impact of these sources of misalignment on the conclusions of an evidence synthesis.

The studies being synthesized may target populations that differ in terms of their baseline characteristics, due to different inclusion/exclusion criteria. Even if inclusion/exclusion criteria are identical, the covariate distribution of the sampled patients will likely vary from study to study. These differences may give rise to heterogeneous treatment effect sizes across studies, which threaten the validity of the evidence synthesis. Random effects models have traditionally been used to account for effect heterogeneity.^24^, ^25^, ^26^ Nevertheless, the conventional approaches do not explicitly produce estimates that are directly interpretable in any particular population.^27^, ^28^, ^29^ This is problematic, given the central role of the *population* in the estimands framework, and in formulating research questions in evidence synthesis and HTA.

Here lies the value of covariate‐adjusted indirect comparisons^20^, ^30^, ^31^ and network meta‐regression approaches,^32^, ^33^ such as those examined by Phillippo et al^2^ in their original simulation study. These methods explicitly account for differences in patient characteristics between studies, thereby targeting estimands in specific samples or populations. As a result, they are being increasingly considered by payers.^34^ In many practical scenarios, patient‐level data will be unavailable for certain trials, and the evidence synthesis will be based on published aggregate‐level data to some degree. Therefore, in the words of Russek‐Cohen, “there needs to be an incentive for summarizing individual trials so they can be more easily studied in meta‐analyses that utilize covariates.”^5^

Conflicts in the summary effect measures that are pooled in an evidence synthesis are also a cause for concern. When the effect measure is non‐collapsible, combining marginal and conditional estimates will produce bias.^12^ Non‐collapsible effect measures such as (log) odds ratios and (log) hazard ratios are commonly used in network meta‐analysis.^35^, ^36^ Finally, there is a need to align within‐study intercurrent event strategies prior to an evidence synthesis.

## 3 CONCLUDING REMARKS

To conclude, I wish to examine some of the considerations made by Senn^7^ and Van Lancker et al^9^ about conditional and marginal estimands. Senn^7^ argues that “we usually treat individuals not populations.” I am in agreement. That being so, reimbursement decisions in HTA are typically made on populations not single patients. At the time of decision‐making, the target population for the therapeutic indication is “stable” and well‐defined, having been prespecified in the PICO question on the scope of the HTA.

The target population of interest is not necessarily broad. Often, it is highly selected and takes into account specific patient characteristics. For instance, the final scope of a health technology appraisal for abemaciclib (TA810), recently published by the National Institute for Health and Care Excellence, describes the target population as “adults with hormone receptor‐positive, HER2‐negative, node‐positive early breast cancer after definitive surgery of the primary breast tumor at high‐risk of recurrence.”^37^

A preference for marginal estimands does not necessarily imply that an “all or nothing” decision is required or that “homogeneity” is assumed. When subsets of the target population are considered to benefit differently from the reimbursement of a drug, relevant sub‐populations are defined during the scoping process, in which case separate PICO questions can be formulated for each. In this case, the covariates used to define the subsets are binary or categorical, and the distinction between marginal and conditional estimands is blurred. A conditional estimand at the population level can be viewed as a marginal estimand at the sub‐population level.

Nevertheless, the specific context—covariate‐adjusted indirect comparisons in HTA—is central to the debate. Consider the situation described by Phillippo et al in their original simulation study:^2^ (1) covariates are continuous; (2) model‐based covariate adjustment is required for transportability; (3) valid estimation relies on correct model specification. As suggested by Spieker,^8^ a “modeling stage” is necessary to select the covariate adjustment model. When the adjustment model is an outcome regression, analysts will seek goodness‐of‐fit to the observed data. In this setting, the conditional estimand would align to the selection of the estimator.

However, the estimands framework demands that the estimand is articulated first, prior to any estimation. Here, the definition of a conditional estimand would depend on statistical modeling assumptions. Conversely, the marginal estimand can be unambiguously interpreted without reference to a particular adjustment model. I believe that this point is implicit throughout Van Lancker et al's discussion.^9^ Ultimately, the marginal estimand is more compatible with the estimands framework, because it is the inferential target that allows one to separate the research question from the method of statistical analysis.
